# Exosome-Mediated Systemic Signaling: Mechanisms, Disease Integration, and Translational Potential

**DOI:** 10.3390/cimb48080845

**Published:** 2026-08-21

**Authors:** Adam Madore, Nigel Walsh, Kush Desai, Gideon Udoh, Naga Gannavaram, Aishniya Kandula, Cohen Yates, Sneha S. Pillai, Komal Sodhi, Bruno S. Goncalves

**Affiliations:** Departments of Surgery, Internal Medicine and Biomedical Sciences, Joan C. Edwards School of Medicine, Marshall University, Huntington, WV 25701, USA; madore1@marshall.edu (A.M.); walsh49@marshall.edu (N.W.); desai25@marshall.edu (K.D.); udoh3@marshall.edu (G.U.); gannavaram@marshall.edu (N.G.); kandula1@marshall.edu (A.K.); yates93@marshall.edu (C.Y.); pillais@marshall.edu (S.S.P.); sodhi@marshall.edu (K.S.)

**Keywords:** exosomes, inter-organ communication, extracellular vesicles, biomarkers, therapeutic targeting

## Abstract

Exosomes have emerged as key mediators of intercellular and inter-organ communication. Although substantial advances have expanded the understanding of the biology of extracellular vesicles, exosome biogenesis and their role in the disease progression of systemic diseases have not yet been fully elucidated. In this review, we present a comprehensive overview of the molecular pathways responsible for exosome biogenesis, emphasizing how the selective incorporation of proteins, lipids, metabolites, messenger RNAs, and microRNAs (miRs) determines the composition and biological activity of exosomes. We also discuss how exosome-mediated inter-organ communication functions as an integrated biological network that connects the kidney, the cardiovascular system, the brain, the liver, the immune system, and tumors, thereby coordinating the pathological responses underlying the progression of chronic diseases. Additionally, we demonstrate the recent advances in the potential of exosomes as minimally invasive biomarkers and clinical translational implantation. Finally, we discussed the methodological and biological challenges that limit the clinical application of exosomes. Overall, this review presents an integrated framework for understanding exosome biology and supports the concept that exosomes function as dynamic platforms for systemic signaling that link molecular mechanisms to disease pathogenesis and translational medicine.

## 1. Introduction

The maintenance of multicellular homeostasis depends on precise communication between distant organs, enabling coordinated physiological responses throughout the body. Traditionally, this communication has been attributed to classical signaling molecules, including hormones, cytokines, and growth factors. Hormones are long-distance signaling molecules that regulate numerous physiological processes, including metabolism, growth, reproduction, and stress responses [1]. Cytokines regulate immune and inflammatory responses by modulating leukocyte activation, differentiation, and intercellular communication [2]. Growth factors regulate cellular proliferation, survival, differentiation, and metabolic adaptation through autocrine, paracrine, and endocrine signaling mechanisms [3]. Although they perform essential biological functions, these molecules exhibit short half-lives, act within restricted spatial and temporal contexts, and rely on concentration gradients to reach their target cells. Consequently, these signaling systems, alone, cannot fully explain the complexity, specificity, and coordinated communication observed during physiological adaptation and chronic disease progression [4,5]. Extracellular vesicles (EVs) have therefore emerged as additional mediators of intercellular communication capable of transferring complex biological information between cells and organs modulating both physiological homeostasis and disease progression [6,7,8,9].

EVs are lipid bilayer particles released by all cells and can be classified based on size, origin, and biogenesis into three groups: apoptotic bodies, microvesicles, and exosomes [10,11]. Apoptotic bodies are relatively large vesicles and are the result of programmed cell death to help clear apoptotic cells, while microvesicles form from outward plasma membrane budding [12]. Exosomes are the smallest of the three and are synthesized through the endosomal pathway, in which intraluminal vesicles form with multivesicular bodies, which are then released from the cell upon fusion with the plasma membrane [13,14]. In accordance with the MISEV2018/2023 recommendations, the term “exosome” is used when referring specifically to vesicles of endosomal origin or when this origin is supported by the cited study; otherwise, the term extracellular vesicles (EVs) is used according to the reported vesicle characteristics. The ability of exosomes to transport functional proteins, lipids, metabolites, and nucleic acids distinguishes them from other extracellular vesicles and has established them as major mediators of intercellular communication [15].

Exosomal cargo is heterogeneous and comprises a wide variety of active molecules including proteins, lipids, messenger RNAs, microRNAs (miRs), DNA fragments, and metabolites [16,17]. This molecular diversity allows exosomes to transmit multiple signals, leading to complex intercellular communication. It has been shown that exosomal proteins have different functions, including signaling molecules, adhesion molecules, and structural proteins related to cargo sorting regulation [18,19,20]. In addition, EV lipids contribute to the vesicle bilayer. They also regulate biogenesis, cargo sorting, EV release, and cellular interactions through lipid-dependent signaling [21,22,23,24]. Furthermore, mRNA transfer between cells via exosomes allows translation in recipient cells and potentially modifies cellular function [15]. Likewise, miRs are among the best-characterized exosomal cargoes. They modulate post-transcription gene regulation in recipient cells and influence cellular phenotype [15].

The recognition of EVs as active mediators of systemic communication has changed our understanding of how biological information is exchanged between cells and organs, both in health and disease. Rather than acting as passive extracellular carriers, sEVs selectively package and deliver bioactive cargo capable of reprogramming gene expression, metabolism, inflammatory responses, and the cellular phenotype in recipient cells, thereby coordinating physiological adaptation or propagating pathological signals to distant tissues. The focus of this review is to discuss the molecular mechanisms underlying exosome biogenesis, the selection of their cargo, and their functional heterogeneity; to explore how EV-mediated signaling coordinates intercellular and inter-organ communication under physiological and pathological conditions; and to highlight their emerging translational potential as biomarkers and therapeutic targets. Overall, this review establishes a conceptual framework in which EVs act as dynamic platforms for systemic signaling that integrate biological processes across various organs and contribute to the onset, progression, and dissemination of complex diseases.

## 2. Exosome Biogenesis, Cargo Selection, and Functional Heterogeneity

The biological properties of exosomes are established through coordinated mechanisms that regulate their biogenesis, cargo selection, and release. These processes ultimately determine exosome composition and contribute to their functional diversity. In addition, exosome populations differ in size, molecular cargo, and biological function, reflecting differences in vesicle biogenesis, donor-cell identity, and the physiological state of the producing cell. Exosome size can vary within the same cell population and may be influenced by the mechanisms involved in vesicle formation and release. Their molecular cargo is also heterogeneous, with differences in the abundance and composition of proteins, lipids, mRNAs, miRs, DNA fragments, and metabolites. This heterogeneity is also reflected in their biological activity, as different exosome populations can have distinct effects on recipient cells depending on their cargo, cellular origin, and target cell. Physiological and pathological conditions can further modify these characteristics by altering exosome biogenesis and cargo selection. As a result, exosomes released by the same cell type may differ in their molecular composition and biological effects. In the following sections, we discuss the molecular mechanisms underlying exosome biogenesis, ESCRT-dependent and ESCRT-independent pathways, selective cargo loading, and the contribution of cellular origin to exosome function.

### 2.1. Endosomal Biogenesis of Exosomes

**Figure 1:**
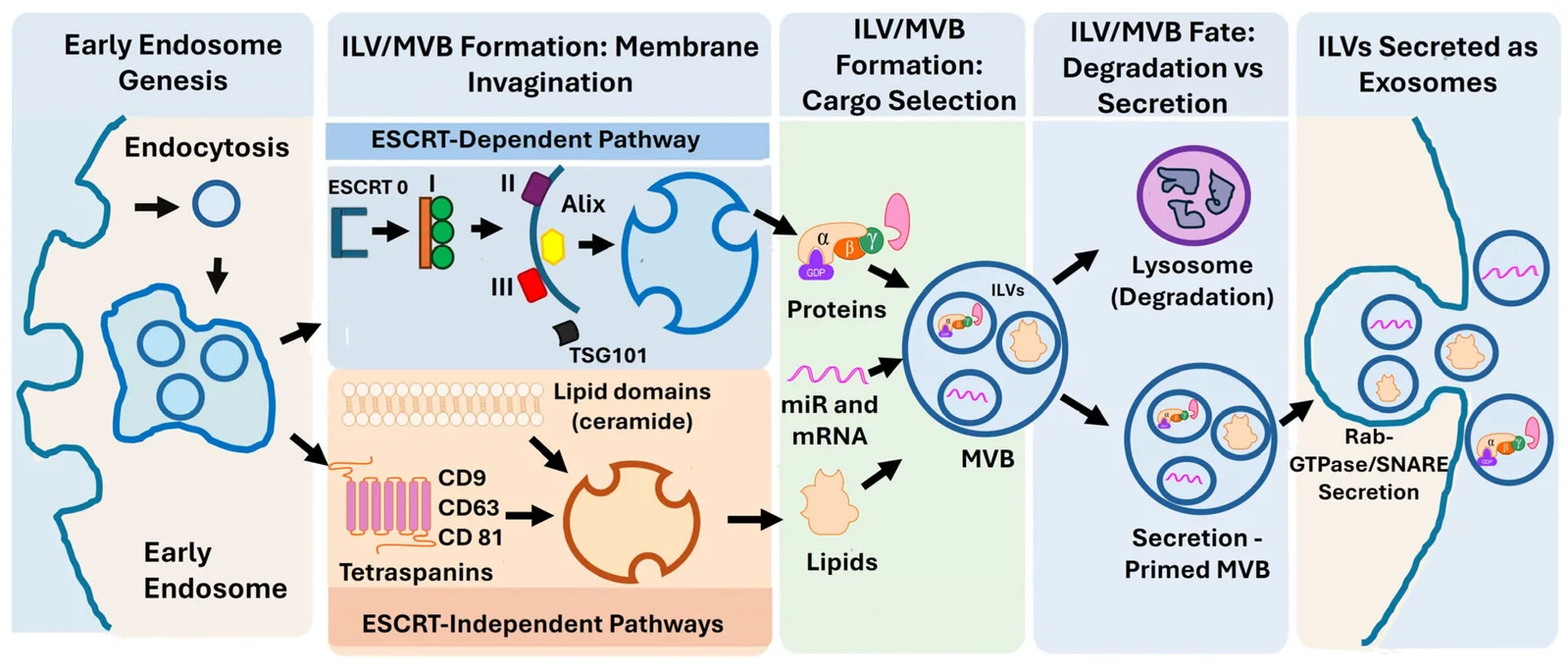
Schematic representation of the molecular mechanisms regulating exosome biogenesis, selective cargo loading, and secretion. Exosome biogenesis begins with early endosome formation, followed by intraluminal vesicle (ILV) generation through endosomal sorting complex required for transport (ESCRT)-dependent and ESCRT-independent pathways. Selective incorporation of proteins, lipids, messenger RNAs (mRNAs), and microRNAs (miRs) determines exosome composition, while multivesicular bodies (MVBs) are subsequently directed toward lysosomal degradation or plasma membrane fusion, resulting in exosome release.

Exosome biogenesis represents the first critical step that determines the composition of sEVs and the biological information that is ultimately transmitted to the recipient cell (Figure 1). Exosome biogenesis begins with endocytic internalization of the cell membrane to produce early endosomes (EEs) [25,26]. EEs contain tubular and vacuolar compartments that sort bioactive cargo, making them determinants of intracellular cargo sorting and packaging [27,28]. As they mature, EE membranes invaginate, forming multiple internal vesicles called intraluminal vesicles (ILVs) that accumulate within multivesicular bodies (MVBs) [29]. Once formed, MVBs either fuse with the lysosome or with the plasma membrane [30]. Fusion of MVBs with the plasma membrane releases intraluminal vesicles into the extracellular space mediated by biomolecules such as MVB and plasma membrane SNARE proteins, Rab GTPases, lipids, and Ca^2+^ where they are subsequently referred to as exosomes [30]. Among Rab GTPases, Rab27a and Rab27b are the best-characterized regulators of MVB docking and exosome secretion, while Rab11 and Rab35 also contribute to exosome release in specific cellular contexts [31,32,33]. Rab proteins act at different stages of endosomal trafficking, and changes in their activity can determine whether MVBs are directed toward secretion or intracellular degradation [34]. Rab27a and Rab27b are recruited to secretory MVBs and facilitate their movement, docking, and subsequent fusion with the plasma membrane [34,35]. Rab11 is mainly associated with recycling endosomes and can regulate the secretion of specific exosome populations, whereas Rab35 participates in MVB trafficking and exosome release in a cell-dependent manner [36,37]. The activity of these GTPases can also change under pathological conditions, resulting in altered exosome secretion and affecting the signals transferred to recipient cells [38]. Endosomal maturation determines exosome cargo composition and subsequent signaling function [39,40]. The process of exosome biogenesis and cargo selection is not fully understood, emphasizing the need for more research on the subject. However, two major pathways indicate that these processes are primarily regulated through endosomal sorting complex required for transport (ESCRT)-dependent and ESCRT-independent pathways [41,42,43].

### 2.2. ESCRT-Dependent Pathways

The ESCRT mechanism is the best-characterized molecular system that regulates exosome biogenesis, coordinating endosomal membrane remodeling with the selective incorporation of the cargo [44]. ESCRT-dependent pathways consist of four complexes (ESCRT-0, -I, -II, -III) together with the ATPase VPS4, responsible for facilitating ILV formation and, most importantly, cargo organization [44]. ESCRT-0 assembles potential ILV cargo by sequestering ubiquitinated biomolecules to EE membranes. ESCRT I and II then inwardly deform the EE membrane while funneling specific biological cargo into the subsequent buds [45]. In addition to promote membrane remodeling, these complexes modulate exosomal composition, with ESCRT-II regulating the incorporation of circRNAs into exosomes [46]. TSG101, a key component of ESCRT-I, mediates the sorting of ubiquitinated cargo into ILVs and contributes to exosome cargo composition [41]. Furthermore, TSG101 modification or knockdown disrupted ILV/MVB formation and the loading of proteins such as CD63 and major histocompatibility complex class (MHC) II [47,48]. Subsequently, ESCRT-III and VPS4 mediate the final membrane remodeling events required for the formation of ILVs, completing the biogenesis of mature ILVs [49,50,51]. At this stage, ESCRT-III interacts with the adaptor protein ALG-2 interacting protein X (ALIX), further facilitating membrane remodeling and the selective incorporation of cargo during vesicle formation [52,53]. As shown above, it consolidates specific biomolecules in ILVs and can alter exosome content when its components are disturbed [54].

### 2.3. ESCRT-Independent Pathways

In addition to ESCRT-dependent mechanisms, ESCRT-independent pathways mediate exosome biogenesis and content without ESCRT complexes [16]. Ceramide promotes endosomal membrane invagination and ILV formation, whereas tetraspanins, including CD9, CD63, and CD81, organize membrane microdomains that facilitate cargo recruitment and vesicle biogenesis [43,55]. It has been shown that changes in ceramide metabolism or in tetraspanin expression alter both exosome secretion and the composition of the molecular cargo [56,57,58]. Beyond ceramide, cholesterol- and phosphatidic acid (PA)-rich domains have been shown to facilitate membrane curvature and cargo selection via interactions with proteins such as Caveolin-1 (Cav-1), syndecan, syntentin, ALIX, and nSMase [59,60,61,62]. Cargo sorting into ILVs can also occur without ubiquitination of the proteins selected for incorporation. In this process, cargo proteins can be recognized through specific protein–protein interactions rather than through a ubiquitin tag. The syndecan–syntenin–ALIX pathway provides one mechanism in which syntenin connects syndecans and associated proteins with ALIX, facilitating their recruitment into ILVs [63]. ALIX can also recognize specific cytoplasmic motifs in membrane proteins, providing another route for ubiquitin-independent sorting that still uses downstream ESCRT components [52]. This mechanism is not limited to a single class of proteins, as MHC class II molecules can be incorporated into exosomes even when ubiquitination sites in their cytoplasmic domains are removed [64]. Thus, ubiquitin-independent sorting increases the range of proteins that can enter ILVs and shows that ubiquitination is not an absolute requirement for cargo selection during exosome formation [52,63,64]. Rather than functioning as independent mechanisms, the dependent and independent ESCRT pathways act in a complementary and context-dependent manner, allowing cells to dynamically regulate exosome biogenesis and the composition of their cargo, depending on their physiological state and cellular origin [31,65,66].

### 2.4. Selective Cargo Loading

Selective cargo loading is a highly regulated biological process that determines the molecular composition and signaling properties of exosomes in response to changes in the cellular microenvironment [41]. Cells actively remodel the contents of exosomes according to their physiological or pathological state, altering the abundance of proteins, lipids, mRNAs, miRNAs (miRs), and other bioactive molecules [16,67]. Selective RNA loading is further regulated by RNA-binding proteins (RBPs) that recognize specific RNA motifs and promote their incorporation into exosomes. Among these, Y-box-binding protein 1 (YBX1) facilitates the selective sorting of miRs, whereas additional RBPs, including Alyref and Fus, recognize sequence-specific EXOmotifs that direct miR load into sEVs [68,69]. Different RBPs recognize distinct RNA sequences, allowing cells to selectively export specific RNAs rather than simply reflecting their intracellular abundance. YBX1, for example, binds miR-223 and promotes its incorporation into exosomes, and the formation of YBX1 condensates further facilitates the recruitment and sorting of this miR [68,70]. SYNCRIP (hnRNPQ) recognizes a specific hEXO motif present in miRs enriched in exosomes, and disruption of SYNCRIP reduces the loading of these miRs [71]. The presence of both EXOmotifs and cellular retention motifs therefore contributes to the distribution of miRs between the cell and released vesicles, with RBPs acting as sequence-specific regulators of this process [69,71]. Among cellular stressors, hypoxia markedly remodels exosomal cargo by increasing proteins, mRNAs, miRs, and lipids including VEGF, VEGFRs and regulatory miRs that influence angiogenesis, inflammation and metabolic adaptation. miR-210 promotes metabolic adaptation by shifting oxidative phosphorylation toward glycolysis and, together with miRs and VEGF-associated proteins, they are able to stimulate angiogenesis through VEGF/VEGFR, Notch/DLL4, and cAMP/PKA signaling [72,73,74,75,76]. Similarly, oxidative stress promotes the selective uptake of inflammatory mediators, damage-associated molecular patterns (DAMPs), cytokines, and stress-responsive RNAs, which amplify inflammatory signaling in recipient cells [77,78]. Furthermore, metabolic disorders, such as obesity and diabetes, together with mitochondrial stress and dysfunction, modify exosome composition, enriching adipokines, miRs, altered insulin signaling proteins, mitochondrial DNA, mitochondrial proteins and lipids, thus reflecting the physiological and pathological state of the donor cell [73,79,80,81,82].

### 2.5. Cellular Origin and Exosome Function

The biological functions of exosomes are determined largely by the identity of the cell of origin, which defines their molecular cargo and, consequently, their signaling properties [83]. Macrophage-derived exosomes promote inflammation activation and inflammation through proinflammatory pathways such as nuclear factor kappa-light-chain-enhancer of activated B cells (NF-κB), thereby increasing immune activation in recipient cells [84,85]. In contrast, endothelial-derived exosomes regulate both vascular homeostasis and endothelial dysfunction. These exosomes disrupt endothelial function by impairing endothelial junction proteins, while endothelial progenitor cell-derived exosomes suppress inflammation, reduce arterial plaques, and promote vascular repair [86,87,88]. However, neuronal exosomes play a role in intercellular communication within the nervous system by transferring regulatory proteins and RNAs that modulate synaptic activity; however, under pathological conditions, they also disseminate neurotoxic proteins, including beta-amyloid and tau protein, thereby contributing to the progression of neurodegeneration [89,90,91,92]. Similarly, tumor-derived exosomes remodel the microenvironments of adjacent and distant tissues, promoting angiogenesis, suppressing antitumor immune responses, and facilitating metastatic spread [93,94,95,96]. These findings demonstrate that functional heterogeneity of exosomes is primarily determined by cellular origin, cargo composition, and the physiological state of the donor cell, allowing exosomes to mediate physiological and pathological processes.

## 3. Exosome-Mediated Cellular Interactions and Signaling Pathways

Exosomes interact with recipient cells through three principal mechanisms: receptor–ligand interactions, direct membrane fusion, and endocytic uptake. Exosomal membrane proteins can interact directly with receptors on target cells, triggering intracellular signaling without the internalization of the vesicles [97,98]. In metastatic melanoma high volumes of exosomes are released that have PD-L1 on their surface which directly binds with PD-1 on CD8+ T cells helping the tumors cells evade apoptosis [95]. Exosomes can also alter lipid metabolism, insulin sensitivity, and glucose uptake via receptor–ligand interactions [99]. Furthermore, exosomes release their molecular cargo through direct membrane fusion or endocytosis. Membrane fusion is mediated by soluble N-ethylmaleimide-sensitive factor attachment protein receptor (SNARE) receptor proteins, such as syntaxin-4, SNAP-23, and VAMP-7, while its efficiency is influenced by extracellular pH, the lipid composition of the membrane, and cholesterol content [100,101]. Alternatively, exosomes can be internalized through several endocytic pathways, including clathrin-mediated endocytosis, micropinocytosis, and phagocytosis [102]. Following membrane fusion or endocytic uptake, exosomes release multiple bioactive molecules, including proteins and RNAs, that act together to regulate recipient-cell function rather than functioning as isolated signaling molecules. Transferred mRNAs are translated after entering recipient cells, where they can influence cellular function [15], and can reach as far as the recipient epigenome, where exosome-packaged DNA methyltransferase mRNAs drive genome-wide hypermethylation [103]. Similarly, exosomal miR-1271-5p suppresses the expression of the SRY-box 6 (SOX6) transcription factor in cardiomyocytes, promoting cell survival and cardiac repair following myocardial infarction [104]. Together, these protein- and RNA-level signals converge on the recipient’s signaling networks and phenotype. Collectively, receptor–ligand signaling, membrane fusion, and endocytic uptake act as complementary routes of communication whose combined output determines the biological response of the recipient cell.

Although exosomes interact with recipient cells through different mechanisms, these interactions initiate intracellular signaling that modulates the biological response of the recipient cell. The biological effects of exosomal communication are ultimately determined by the activation of interconnected signaling pathways that regulate inflammation, metabolism, proliferation, survival, and tissue remodeling. Among these, NF-κB represents a key inflammatory hub, frequently activated by the exosomal cargo. Activated microglia exosomes can promote other microglia activation by transmitting polarized signals to resting M0 microglia using miR-155-5p which activates the NF-κB pathway, causing an inflammatory cascade [105]. In addition to directly activating NF-κB signaling, exosomal cargo can modulate phosphoinositide 3-kinase/protein kinase B (PI3K/AKT) signaling, highlighting the extensive crosstalk between inflammatory and cell survival pathways [106]. Exosomal miR-143-3p suppresses the PI3K/AKT pathway, promoting M1 macrophage polarization while simultaneously enhancing NF-κB activation in periodontitis [107]. However, exosomal miR-486-5p derived from bone marrow stromal cells inhibits phosphatase and tensin homolog deleted on chromosome 10 (PTEN), thereby activating the PI3K/AKT signaling pathway and protecting cardiomyocytes from apoptosis [108]. These pathways further converge on transcription factor 3 (STAT3), amplifying the downstream consequences of exosomal signaling. In addition, exosomal high mobility group box 1 (HMGB1) activates the toll-like receptor 4 (TLR4)/NF-κB/STAT3 axis, promoting tumor growth, immune suppression, and M2 macrophage polarization [109]. In contrast, EV-mediated STAT3 signaling can promote the transition of astrocytes from the neurotoxic A1 phenotype to the neuroprotective A2 phenotype, highlighting the context-dependent effects of STAT3 activation [110]. In addition, persistent inflammatory signaling also involves the transforming growth factor-beta (TGF-β)/SMAD pathway, linking inflammation to fibrotic remodeling [111,112]. These signaling networks also interact with the NLR family inflammasome containing the pyrin domain 3 (NLRP3), generating self-amplifying inflammatory circuits. Exosomal miR-21 increases the production of tumor necrosis factor alpha (TNF-α), resulting in the activation of STAT3, which, in turn, further enhances the expression of miR-21, thereby sustaining chronic inflammatory signaling through a positive feedback loop [113,114].

Exosomes function as systemic inflammatory signal propagators by converting localized tissue injuries into wide, systemic inflammatory responses. They achieve this by releasing various inflammatory miRs, cytokines, transcription factors, and by penetrating biological barriers [115]. Serum-derived exosomes isolated from septic mice localize in the lungs of healthy recipient mice and induce acute pulmonary inflammation, providing direct evidence that circulating exosomes propagate inflammatory signals to distant organs [116]. Persistent EV-mediated communication further promotes chronic low-grade inflammation through self-perpetuating inflammatory circuits. Senescent cells continuously release exosomes enriched in miR-30b-5p, which activate NF-κB signaling and increase the production of interleukin (IL)-1β and IL-6, while simultaneously carrying an immunosuppressive cargo that limits the elimination of senescent cells, thereby perpetuating chronic inflammation [117,118]. In addition, it has been shown that in metabolic diseases sonic hedgehog (Shh)-positive exosomes promote insulin resistance by modulating hormone-sensitive lipase, while exosomes containing retinol-binding protein 4 (RBP4) stimulate the production of TNF-α and IL-6 by macrophages in obesity [119,120]. The persistent spread of inflammatory signals ultimately contributes to multi-organ dysfunction. EVs isolated from patients with chronic kidney disease (CKD) induce cardiomyocyte apoptosis, impair contractile function, and disrupt calcium homeostasis. Moreover, reducing circulating exosomes improves cardiac function in experimental models, suggesting that kidney-derived exosomes carrying cardiotoxic miRs contribute to cardiorenal dysfunction [7]. The signaling pathways discussed above, including NF-κB, STAT3, and NLRP3, also regulate metabolic homeostasis. This common signaling network links exosome-mediated inflammation to the metabolic alterations observed in chronic diseases.

Exosomes play a central role in metabolic signaling, coordinating communication between adipose tissue, the liver, skeletal muscle, and the cardiovascular system, thereby regulating insulin sensitivity, redox homeostasis, and cellular adaptation to metabolic stress. Exosomes derived from adipose tissue macrophages of obese mice induce insulin resistance in healthy recipients through the transfer of miR-155, whereas exosomes from lean mice improve glucose tolerance and insulin sensitivity without altering body weigh [121]. Exosomal miRs further regulate metabolic homeostasis through complementary mechanisms. Increased miR-34a suppresses M2 macrophage polarization, promoting a pro-inflammatory phenotype associated with insulin resistance [122], while miR-155 inhibits suppressor of cytokine signaling 1 (SOCS1), favoring M1 polarization. Similarly, elevated levels of exosomal miR-122, miR-192, and miR-27a/b-3p contribute to the propagation of insulin resistance in obesity [123]. Exosomes also promote oxidative stress through the NADPH oxidase (NOX) system by transferring mitogen-activated protein kinase 1 and 2 (MEK1/2) and extracellular signal-regulated kinases 1 and 2 (ERK1/2) to recipient cells, thereby activating MEK/ERK signaling and increasing NOX4 expression. Additionally, exosomes can directly transfer functional NOX2 to recipient cells, further increasing superoxide production and redox imbalance [124]. Cellular nutritional status further regulates both exosome biogenesis and the composition of their contents. In nonalcoholic fatty liver disease, hepatocyte-derived EVs are enriched in palmitic and stearic acids, activating TLR4 signaling and impairing insulin sensitivity [125]. Similarly, nutrient availability regulates EV secretion via the mammalian target of rapamycin complex 1- SCY1-like pseudokinase 1 (mTORC1-SCYL1) axis; nutrient deprivation increases exosome release, while excess nutrients suppress the secretion of extracellular vesicles [126]. The principal mechanisms underlying exosome-mediated signaling and their major biological consequences are summarized in Table 1.

## 4. Exosome Remodeling Under Pathological Conditions

Pathological conditions profoundly reshape the biogenesis, cargo composition, and biological function of exosomes. Rather than simply increasing exosome release, disease-associated cellular stress actively restructures the molecular cargo packaged within these vesicles, thereby altering their signaling properties and functional effects on recipient cells. Consequently, pathological exosomes become active mediators of systemic communication, amplifying inflammation, metabolic dysfunction, and tissue damage in various organs. Although diabetes, chronic kidney disease, and cardiovascular disease are distinct disorders, exosome-mediated remodeling in these conditions shares several common features, including chronic inflammation, oxidative stress, and altered intracellular signaling, all of which contribute to disease progression.

### 4.1. Diabetes-Associated Remodeling

Chronic hyperglycemia is one of the best-characterized examples of pathological exosome remodeling. Chronic hyperglycemia induces widespread cellular signaling abnormalities, including activation of the advanced glycation end-product–receptor for advanced glycation end-products (AGE-RAGE) axis, PKC signaling, the hexosamine biosynthetic pathway, and the polyol pathway, collectively promoting oxidative stress, inflammation, and endothelial dysfunction [128,129,130]. These metabolic disturbances also remodel exosome-mediated communication; under conditions of hyperglycemia, cells secrete exosomes enriched with pathogenic proteins and regulatory RNAs, including components of the renin–angiotensin system and pro-inflammatory miRs, such as miR-155, which promote insulin resistance, inflammation, and tissue dysfunction in recipient cells [121]. A major downstream effect of this glucotoxicity is oxidative stress in which elevated reactive oxygen species (ROS) levels influence the sorting of miRs, proteins, and lipids into exosomes, encouraging the release of exosomes with upregulated with pro-inflammatory, pro-apoptotic, and profibrotic pathways [131,132]. Additionally, diabetic exosomes contribute directly to such vascular injury by transferring pathogenic miRs and inflammatory mediators to endothelial cells, amplifying the effects of hyperglycemia induced oxidative stress and endothelial dysfunction. Exosomes derived from AGE-stimulated macrophages, enriched with miR-22-5p, suppress Forkhead box P1 (FOXP1) signaling, leading to increased endothelial inflammation, decreased migration, and reduced reparative capacity [133]. In addition to their direct effects on endothelial cells, diabetic exosomes further amplify inflammatory signaling by promoting macrophage activation, NF-κB signaling, and NLRP3 inflammasome activation [85]. Furthermore, hyperglycemic macrophages release exosomes enriched with miR-500a-5p, which suppresses anti-inflammatory pathways and enhances NLRP3 inflammasome activation, in turn increasing production of cytokines such as IL-1β, IL-6, and TNF-α [134].

### 4.2. Chronic Kidney Disease

CKD modulates exosome-mediated intercellular communication, enabling the propagation of pathological signals that contribute to both renal and cardiovascular complications [135]. As kidney function deteriorates, the accumulation of uremic toxins including p-cresyl sulfate, fibroblast growth factor 23, AGEs, indoxyl sulfate (IS), uric acid, and inflammatory cytokines alters the composition of exosomes and their signaling properties [136,137,138]. In addition, uremic toxins activate PI3K/AKT signaling, increasing exosomal surface glycosaminoglycan (GAG) expression which alters ligand-binding interactions [134]. Exposure to IS further alters the composition of EVs, increasing the levels of proteins associated with adipogenesis, inflammatory signaling, and xenobiotic metabolism, while reducing the proliferation of endothelial cells [139]. CKD-associated EVs further impair angiogenesis by increasing matrix metallopeptidase 1 (MMP1) and cathepsin L (CTSL) expression and depleting pro-angiogenic miR-130a-3p and miR-126-3p, resulting in defective vascular repair and endothelial dysfunction. They also enhance intercellular adhesion molecule 1 (ICAM-1) and vascular cell adhesion molecule-1 (VCAM-1) expression, leukocyte recruitment, and NLRP3IL-1β-IL-6 signaling which creates a self-amplifying cycle of inflammation and oxidative stress [139,140,141,142,143,144]. In addition to vascular injury, exosomes derived from CKD promote profibrotic remodeling via TGF-β, miR-21, the PTEN/AKT and Smad7 pathways, while additional pathways involving Shh, the β-catenin-osteopontin (OPN)–CD44 axis, and p53 degradation mediated by tumor necrosis factor alpha-induced protein 8 (TNFAIP8) further amplify extracellular matrix deposition [145,146,147,148,149]. These pathological changes extend beyond the kidney, as exosomes derived from CKD induce cardiomyocyte apoptosis, impair cardiac function, and promote vascular calcification by reducing fetuin-A, Gla-rich protein, and miRs that regulate VEGFA, thereby leading to the osteogenic transformation of vascular smooth muscle cells and cardiorenal dysfunction [7,150,151,152].

### 4.3. Cardiovascular Disease

Cardiovascular disease (CVD) describes a broad group of heart pathologies all resulting in vascular injury and cardiomyocyte stress [153]. Under inflammatory and hypoxic conditions, cardiomyocytes increase exosome secretion through the activation of the NLRP3 inflammasome, the mechanistic target of rapamycin (mTOR), and the endosomal sodium–proton exchanger NHE9, while simultaneously enriching the exosomal cargo with hypoxia-inducible factor 1-alpha (HIF-1α) and Hsp70, thereby reflecting the cellular response to stress [154,155]. These stress-induced exosomes further regulate tissue remodeling through bioactive proteins and miRs. Among heat shock proteins (Hsps), Hsp20 activates the AKT signaling pathway and attenuates inflammation, while Hsp60 stimulates TLR4-dependent cytokine production, Hsp70 exerts protective effects during ischemia, and Hsp90 promotes collagen synthesis by activating the signal transducer and activator of STAT3 [156,157,158]. In addition to cardiomyocyte remodeling, cardiovascular exosomes disrupt vascular homeostasis by promoting endothelial activation and inflammatory signaling. EVs derived from atherosclerotic plaques suppress dual-specificity protein phosphatase 5 (DUSP5), activate mitogen-activated protein kinase (MAPK) signaling, and increase the expression of ICAM-1, VCAM-1, and E-selectin, while exosomes derived from cardiomyocytes impair endothelial vascular relaxation; furthermore, aging is associated with an increase in circulating endothelial-derived EVs, which supports their contribution to endothelial dysfunction [159,160,161]. These vascular changes also amplify inflammatory responses via TLR4, RAGE, and the NLRP3-IL-1β axis, highlighting inflammation as a central factor in the progression of atherosclerosis [159,160,162,163]. Persistent inflammatory signaling leads to myocardial fibrosis through multiple exosome-mediated pathways, such as AKT, PTEN, miR-217, Peli1, NF-κB, Shh/Gli1, extracellular signal-regulated kinase (ERK)/MAPK, and the TGF-β/Smad2/3 cascade, leading to the deposition of collagen I/III, myofibroblast activation, myocardial stiffening, and progressive cardiac dysfunction [164,165,166].

### 4.4. Pathological Reprogramming

**Figure 2:**
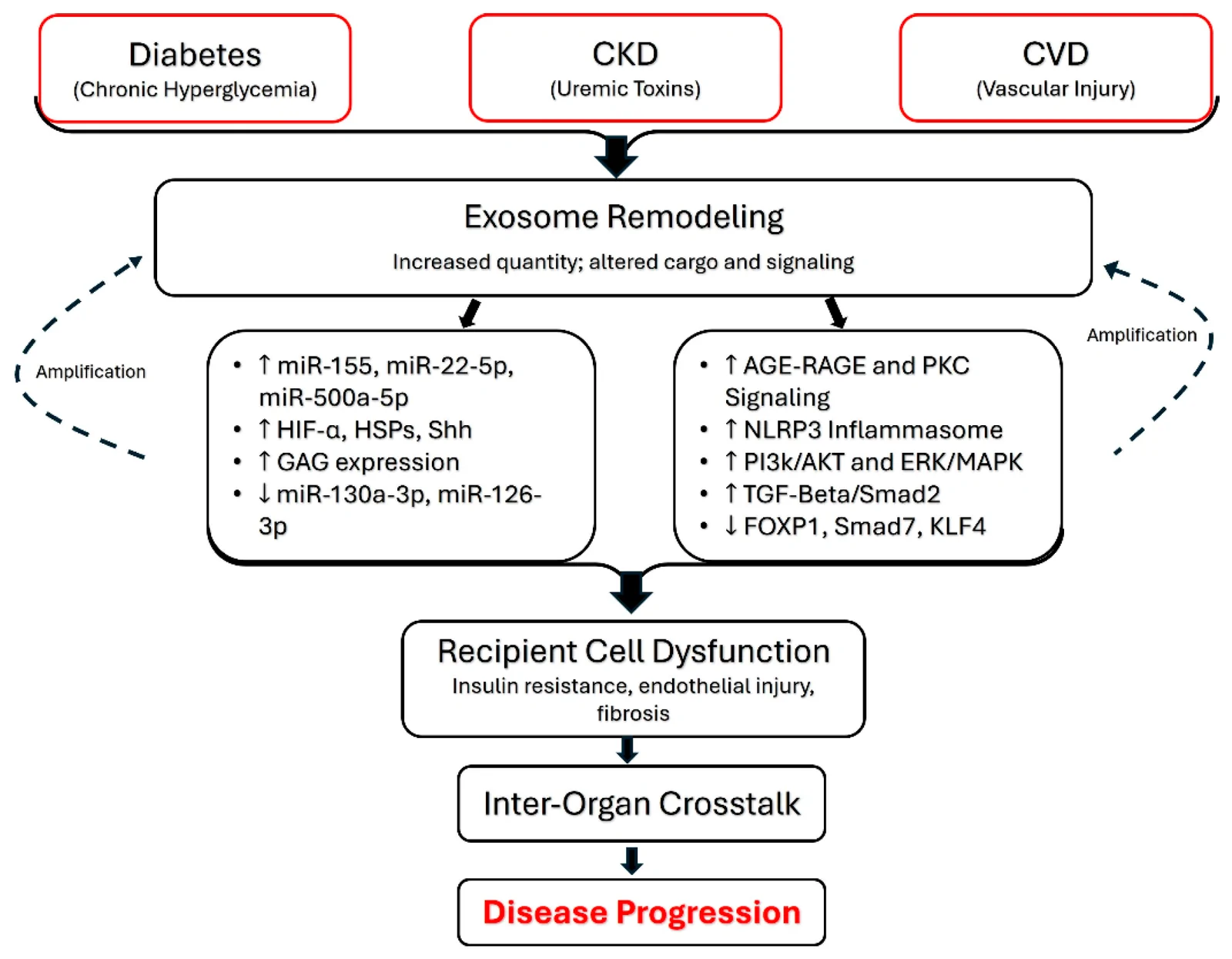
Schematic representation showing exosome remodeling during chronic diseases. Disease-specific pathological stimuli alter exosome biogenesis, molecular cargo, and signaling properties, promoting recipient-cell dysfunction, inter-organ communication, and disease progression. Abbreviations: HIF-1α, hypoxia-inducible factor-1α; HSPs, heat shock proteins; Shh, Sonic hedgehog; GAGs, glycosaminoglycans; AGE-RAGE, advanced glycation end-product–receptor for advanced glycation end-products; NLRP3, NLR family pyrin domain containing 3; PI3K/AKT, phosphoinositide 3-kinase/protein kinase B; ERK/MAPK, extracellular signal-regulated kinase/mitogen-activated protein kinase; TGF-Beta, transforming growth factor-β; FOXP1, forkhead box protein P1; and KLF4, Krüppel-like factor 4.

As discussed to this point, pathological microenvironments reshape exosome composition and function (Figure 2). Varying stressors throughout the body trigger the modulation of both the quantity of and composition within exosomes. Under hypoxic stress, cells release greater numbers of exosomes while simultaneously altering their molecular cargo to include factors such as HIF-1α, Hsps (including Hsp70), and molecules that promote angiogenesis. This remodeling process is regulated through several interconnected pathways, including HIF-1α-dependent transcriptional programs, RAB family GTPases, and reactive oxygen species-driven mechanisms [155]. Furthermore, oxidative stress induces a widespread remodeling of the mRNA content of exosomes, demonstrating that pathological microenvironments actively reprogram the molecular information transferred between cells [167]. Tumor-derived exosomes reprogram distant tissues to establish pro-metastatic niches [168]. Similarly, circulating exosomes derived from chronic liver diseases promote fibrosis through miR-mediated activation of profibrotic pathways, including the suppression of Krüppel-like factor 4 (KLF4), thereby extending pathological remodeling beyond the primary site of the lesion [169]. These findings explain how rather than serving as passive biomarkers of tissue injury, exosomes have emerged as active participants in disease propagation. A common feature across chronic pathological states is the establishment of a feed-forward positive feedback cycle in which stressed microenvironments secrete exosomes which continuously influence the behavior of both surrounding and distant tissues. This ability to coordinate pathological communication across multiple organs has emerged as a central mechanism underlying the systemic integration of chronic diseases. Thus, disease-associated remodeling of exosomes extends the effects of local cellular stress beyond the tissue of origin. Once released into the circulation, these altered vesicles can transmit pathological signals to distant organs, establishing the inter-organ communication networks discussed in the following section.

## 5. Inter-Organ Communication and Disease Integration

Inter-organ communication is essential for maintaining physiological homeostasis and coordinating adaptive responses to environmental and pathological stimuli [170]. Although endocrine, neural, and immunological pathways have long been recognized as the primary mediators of systemic communication, growing evidence indicates that exosomes constitute an additional layer of biological regulation by transporting regulatory proteins, lipids, metabolites, and nucleic acids between distant organs [171,172]. This exosome-mediated exchange allows tissues to influence the function of distant target organs, under both physiological and pathological conditions, thereby coordinating inflammatory, metabolic, fibrotic, and immunological responses at the organismal level. The following sections illustrate how exosome-mediated communication integrates the kidney-cardiovascular axis, the gut–brain axis, and tumor–host interactions, highlighting its broader role as a systemic signaling network that drives the progression of chronic diseases.

### 5.1. Kidney to Cardiovascular Axis

The kidney and the cardiovascular system are closely linked through bidirectional signaling pathways, and EVs have emerged as important mediators of the cardiorenal axis [7]. EVs derived from CKD propagate pathological signals that contribute to fibrosis, vascular calcification, endothelial dysfunction, and, ultimately, cardiorenal syndrome. Cardiac fibrosis is associated with CKD, and the miRs in EVs associated with CKD have been shown to be correlated with proven markers of cardiac injury. Removal of CKD EVs ameliorated adverse cardiac remodeling, showing kidney-derived EVs drive negative cardiac remodeling, including fibrosis [7,173]. Vascular calcification is a common complication in patients with CKD, resulting from the accumulation of calcium phosphate salts in the medial and intimal layers of the vessel wall. Elevated calcium levels increase exposure to phosphatidylserine and the accumulation of annexin A6, leading to the formation of nucleation sites for hydroxyapatite deposition, while a reduction in fetuin-A further promotes mineralization [151,174,175]. Calcifying EV release is also modified by signaling pathways such as epidermal growth factor receptor (EGFR), IκB kinase β (IKK2)/NFKB, and proprotein convertase subtilisin/kexin type (PCSK8) all involved in vascular smooth muscle cell (VSMC) apoptosis and EV biogenesis [176,177,178,179]. In addition to vascular calcification, EVs derived from CKD impair endothelial integrity through uremic toxin-induced remodeling of exosomal content. Indoxyl sulfate alters the composition of exosomes, reducing endothelial proliferation and promoting endothelial dysfunction [139]. Furthermore, exosomes isolated from CKD patients on dialysis induced endothelial–mesenchymal transition (EndMT), reducing the endothelial markers CD31 and vascular endothelial cadherin, as well as increasing vimentin and N-cadherin [180]. Additionally, reduced levels of circulating miR-126 and altered EV miR profiles contribute to endothelial dysfunction in patients with CKD and coronary artery disease [140,181]. In addition to mediating inter-organ interactions, EV miR signatures have emerged as promising biomarkers of adverse cardiac and renal outcomes in CKD [7,8,182].

### 5.2. Gut–Brain Axis

The gut microbiota communicates with distant organs not only through soluble metabolites but also through EVs that carry bioactive molecules capable of modulating host signaling pathways [183,184]. Microbial metabolites can influence signaling via metabolites within microbiota-released EVs, which have been shown to contain many bioactive materials such as gabapentin, glutamate, arachidonoyl-dopamine, and N-acetlycatecholamines. Importantly, these EVs have been shown to cross intestinal and blood–brain barriers, allowing the influence of distant organs and the gut–brain axis [185]. In individuals with compromised gut barriers, lipopolysaccharide (LPS)-positive bacterial EVs in plasma can be isolated and are able to induce systemic immune activation [186]. Similarly, diarrheal microbiota-derived EVs induce a macrophage polarization compromising epithelial barrier integrity; furthermore, these diarrheal microbiota-derived EVs inhibited NF-KBIA, increasing macrophage NFKB signaling, driving inflammation [187].

### 5.3. Tumor to Systemic Metabolism

Tumor-derived exosomes (TDEs) act as potent mediators of inter-organ communication, reprogramming metabolism and immune responses in distant tissues [188,189,190]. One of the best-characterized systemic consequences is cancer cachexia, in which TDEs promote the loss of skeletal muscle mass through the activation of apoptotic and catabolic pathways mediated by exosomal miRs, growth and differentiation factor 15 (GDF-15), and HsPs [191,192,193]. In addition to muscle atrophy, TDEs and EVs induce profound metabolic remodeling by altering glucose metabolism, insulin sensitivity, oxidative phosphorylation, protein synthesis, and ketone body oxidation in skeletal muscle, while simultaneously reprogramming metabolic pathways in the brain, lungs, and pancreas [194,195,196,197,198,199,200]. These metabolic effects are mediated, in part, by miRs and exosomal and EV lipids that impair glucose uptake, inhibit pyruvate kinase, and promote inflammatory signaling [194,195,201]. Furthermore, TDEs establish an immunosuppressive microenvironment by inhibiting CD8^+^ T cells and natural killer (NK) cells, while promoting M2 macrophage polarization, the expansion of regulatory T cells (Tregs), and the accumulation of myeloid-derived suppressor cells (MDSCs) [202,203,204,205,206].

### 5.4. Disease Integration

**Figure 3:**
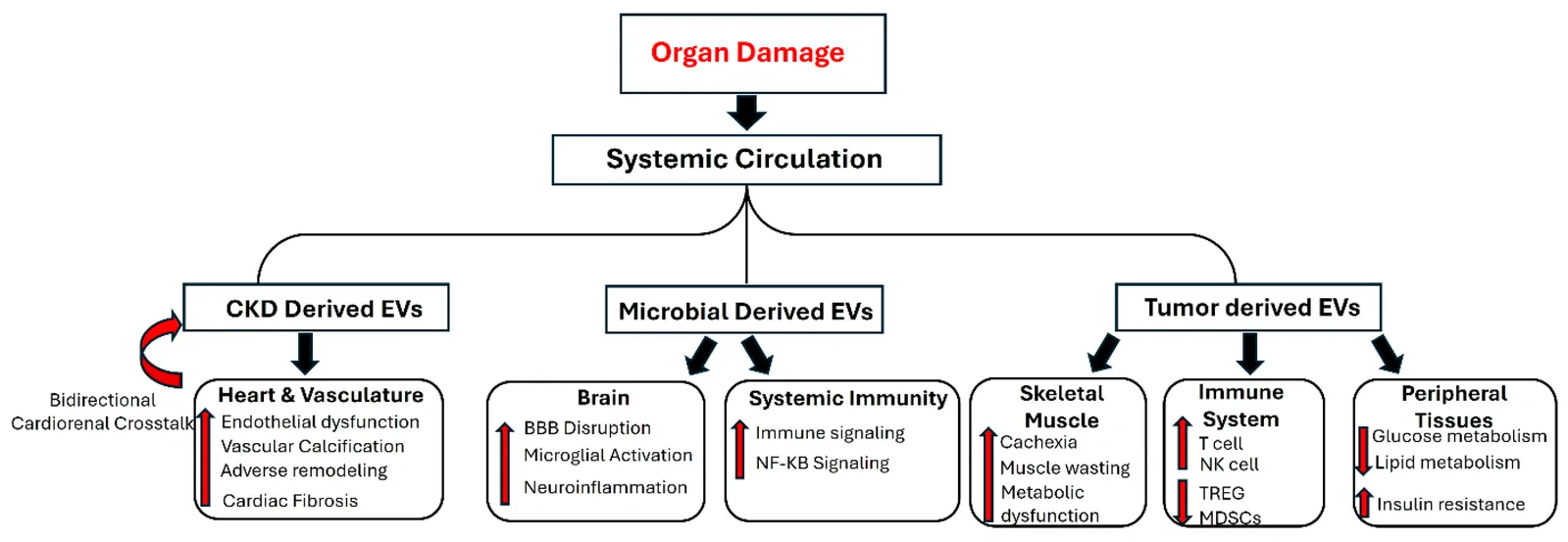
Schematic representation showing exosome-mediated inter-organ communication during chronic diseases. Disease-associated exosomes released into the systemic circulation establish pathological crosstalk between distant organs, promoting cardiovascular dysfunction, neuroinflammation, metabolic remodeling, immune dysregulation, and disease progression. Abbreviations: BBB, blood–brain barrier; NF-κB, Nuclear Factor Kappa B; T cells, CD8^+^ T cells; NK, natural killer cells; Tregs, regulatory T cells; MDSCs, myeloid-derived suppressor cells.

Exosomes establish dynamic communication networks between organs that allow pathological signals originating in one tissue to influence the function of distant organs, thereby coordinating the progression of systemic disease (Figure 3). Although the cardiorenal axis, gut–brain axis, and tumor–host interactions involve distinct organs and pathological conditions, they share a common mechanism in which exosomes and EVs transfer proteins, lipids, metabolites, and nucleic acids that modulate signaling pathways involved in inflammation, fibrosis, metabolism, and immune responses. Through these shared mechanisms, pathological signals are propagated and amplified across multiple organs, contributing to disease. The cardiorenal axis is a representative example, in which kidney-derived exosomes and EVs promote cardiovascular dysfunction through fibrosis, vascular calcification, endothelial injury, and adverse remodeling, while reciprocal signaling from the damaged heart further contributes to renal damage, establishing a bidirectional communication network [7,8,139,207]. Similarly, microbiota-derived exosomes and EVs link intestinal dysfunction to systemic immune activation and neuroinflammation by crossing biological barriers and modulating NF-κB signaling, IL-6 production, blood–brain barrier integrity, and microglial activation [208,209,210]. Tumor-derived exosomes, in turn, lead to systemic metabolic remodeling by promoting skeletal muscle cachexia, suppressing the activity of CD8^+^ T cells and NK cells, expanding Tregs and MDSCs, and altering glucose and lipid metabolism in peripheral tissues [121,189,194,195,204,206]. These all show that exosomes, through coordinating pathological inter-organ signaling, can act as systemic integrators of disease [211,212,213,214]. The molecular changes associated with this systemic communication can also be detected in circulating exosomes and EVs, providing information about disease activity across different organs. These characteristics support their use as biomarkers for disease detection and monitoring and provide a basis for therapeutic targeting, as discussed in the following section.

## 6. Translational Applications: Biomarkers and Therapeutic Targeting

The presence of exosomes and EVs in biological fluids has established them as promising biomarkers for the minimally invasive detection and monitoring of diseases. Exosomes can be found in all types of tissue and have the potential to be isolated from various bodily fluids, including urine, plasma, saliva, and cerebrospinal fluid (CSF), among others, making them attractive candidates for liquid biopsy applications [215,216,217,218]. This makes exosomes and EVs attractive candidates for minimally invasive disease monitoring. It is important to note that the molecular signature supports the diagnostic value of exosomes and also provides information about disease mechanisms, creating opportunities for the development of targeted therapies. Their therapeutic potential ties back to the highly specific cargo they hold. Despite these advantages, the immunogenic profile of exosomes and EVs should also be considered when evaluating their therapeutic potential. Although exosomes and EVs generally exhibit low immunogenicity, immune recognition can vary according to their cellular source, surface composition, molecular cargo, and manufacturing process [219]. Exosomes and EVs can carry immune-related molecules from their cells of origin, including major histocompatibility complex (MHC) molecules, while proteins, lipids, and nucleic acids within or on the surface of the vesicles can also affect innate and adaptive immune responses [219,220,221,222]. The purity of exosome and EV preparations is another important factor, since co-isolated proteins, lipoproteins, or other extracellular components may contribute to unwanted immune responses [223]. These issues have direct clinical implications as immune recognition may affect the clearance, biodistribution, therapeutic activity, and the safety of repeated administration [219,224]. Current clinical evidence is encouraging, with a recent meta-analysis reporting a low incidence of serious adverse events following EV-based therapies; however, substantial differences in EV source, isolation, characterization, and administration among clinical studies still limit conclusions regarding their long-term safety [225]. Strategies to reduce immunogenicity include careful selection of the donor-cell source, rigorous purification and characterization of EV preparations, and optimization of dose and administration protocols [219,223]. Modification of the parental cells or EV surface to reduce immune recognition is another approach being investigated for therapeutic applications [219,226]. Therefore, careful characterization of exosome and EV populations remains an important step for the safe and effective development of exosome-based therapies [222,227].

Biomolecules such as proteins, lipids, and miRs are all found within secreted exosomes and represent not just the cell of origin but also the disease status of the originating tissue. This provides a overview into the health status of various tissues. These danger signals often manifest long before clinical symptoms may appear [228,229,230]. The evidence supporting the diagnostic utility of exosomal content has been growing in various disease contexts. In neurodegenerative diseases exosomal miR signatures have been associated with Alzheimer’s disease up to 5 to 7 years before the onset of cognitive symptoms [228]. Similarly, circulating EV miRs have demonstrated high diagnostic accuracy for the early detection of gastric cancer [219]. Additionally, altered exosomal miR profiles have also been associated with diabetic kidney disease and cardiovascular complications [231,232,233,234]. Furthermore, exosomal content also provides prognostic information. Exosomal miR profiles predict overall survival and progression-free survival in patients with colorectal cancer undergoing anti-EGFR therapy, while the exosomal miRs miR-23b-3p, miR-10b-5p, and miR-21-5p significantly improved survival prediction in non-small cell lung cancer [235,236]. These studies represent the growing utility of exosomes as biomarkers of disease status, diagnostic potential, and prognostic value.

Among the biological fluids currently used for exosome and EV isolation, plasma is the most clinically relevant source due to its routine collection, minimal invasiveness, and ability to reflect systemic physiological and pathological processes [237]. Circulating exosomes originate from various tissues, allowing their molecular cargo to provide a dynamic view of inter-organ communication during both acute and chronic diseases. This systemic signaling has been demonstrated in various pathological conditions. During sepsis, plasma-derived exosomes are upregulated in levels of cytokines and chemokines, improving T cell differentiation and perpetuating the inflammatory disease state far from the cell of origin [116]. Similarly, exosomes isolated from diabetic mice induce diabetic-like conditions when introduced into non-glucotoxicity cells, while nondiabetic exosomes injected into obese mouse models preserve insulin sensitivity, demonstrating that circulating exosomes actively mediate systemic metabolic communication [121]. The dynamic nature of exosomal content also supports its use in the longitudinal monitoring of disease. In patients with head and neck cancer, levels of circulating exosomal proteins decrease during remission and increase after disease recurrence [238], while levels of the human endogenous retrovirus type W exosomal envelope protein (pHERV-W ENV) predict disease activity and progression in multiple sclerosis [239]. Furthermore, the remarkable stability of exosomal RNA allows for the long-term storage of samples without significant degradation, facilitating both prospective monitoring and retrospective molecular analyses [240]. Although these findings highlight the considerable clinical potential of plasma-derived exosomes, several technical and biological challenges currently limit their routine implementation as reliable clinical biomarkers. Representative clinical applications of exosomal biomarkers are summarized in Table 2.

Despite their considerable translational potential, several methodological and technical challenges continue to limit the clinical implementation of exosome- and EV-based biomarkers. Variability among isolation methods remains a major source of inconsistency, as different approaches yield exosome populations that differ in yield, purity, and molecular composition. Affinity-based methods, such as SubX, yield highly purified vesicles with relatively low recovery, while ultracentrifugation provides higher yields but lower purity [255]. Sample purity poses an additional challenge, as EV preparations often contain protein aggregates, lipoproteins (LPPs), and other EVs with overlapping biophysical properties [256]. Even purification strategies that combine size-exclusion chromatography with density gradients do not eliminate contamination by LPPs, while the identification of physiological complexes between EVs and LPPs further complicates the distinction between contaminating particles and biologically relevant interactions [257,258]. Standardization remains another major obstacle, as preanalytical variables including sample collection, storage conditions, transport, centrifugation protocols, and analytical workflows substantially influence the composition of EVs and subsequent analyses [259]. Although international guidelines for the characterization of EVs have substantially improved reporting practices [260,261], harmonized methodologies and rigorous interlaboratory validation remain prerequisites for the routine clinical implementation of exosome- and EV-based biomarkers. The same factors that influence the reliability of exosome- and EV-based biomarkers also influence the development of therapeutic applications, since isolation methods, purity, and manufacturing procedures directly determine the composition and biological activity of the vesicles.

Exosomes and EVs have emerged as promising translation platforms due to their dual capacity to serve as minimally invasive biomarkers and biological therapeutic carriers. Their molecular cargo reflects the physiological and pathological state of the cell of origin, whereas their intrinsic ability to transfer functional proteins, lipids, and regulatory RNAs provides opportunities for targeted therapeutic intervention. Clinical studies have already demonstrated the diagnostic potential of exosomal miR signatures for early disease detection, including gastric cancer and multiple sclerosis, while preclinical investigations have shown that mesenchymal stem cell-derived exosomes improve cardiac repair by reducing mitochondrial dysfunction and oxidative stress through activation of the AMP-activated protein kinase–peroxisome proliferator-activated receptor gamma coactivator 1-alpha (AMPK/PGC-1α) signaling pathway [230,244,262]. However, successful clinical translation requires substantially more than technological advances alone. Differences in isolation strategies, manufacturing platforms, and exosome- and EV-producing cell populations continue to influence vesicle composition and biological activity, as illustrated by the distinct miR profiles obtained using different isolation methods and the functional heterogeneity observed between Y201- and Y202-derived mesenchymal stem cell exosomes and EVs [241,245,263]. Future progress will therefore depend on integrating rigorous mechanistic validation of exosomal cargo with standardized manufacturing procedures, harmonized analytical methodologies, and multicenter clinical validation, thereby establishing reproducible exosome-based biomarkers and therapeutics suitable for routine clinical practice.

## 7. Conclusions

**Figure 4:**
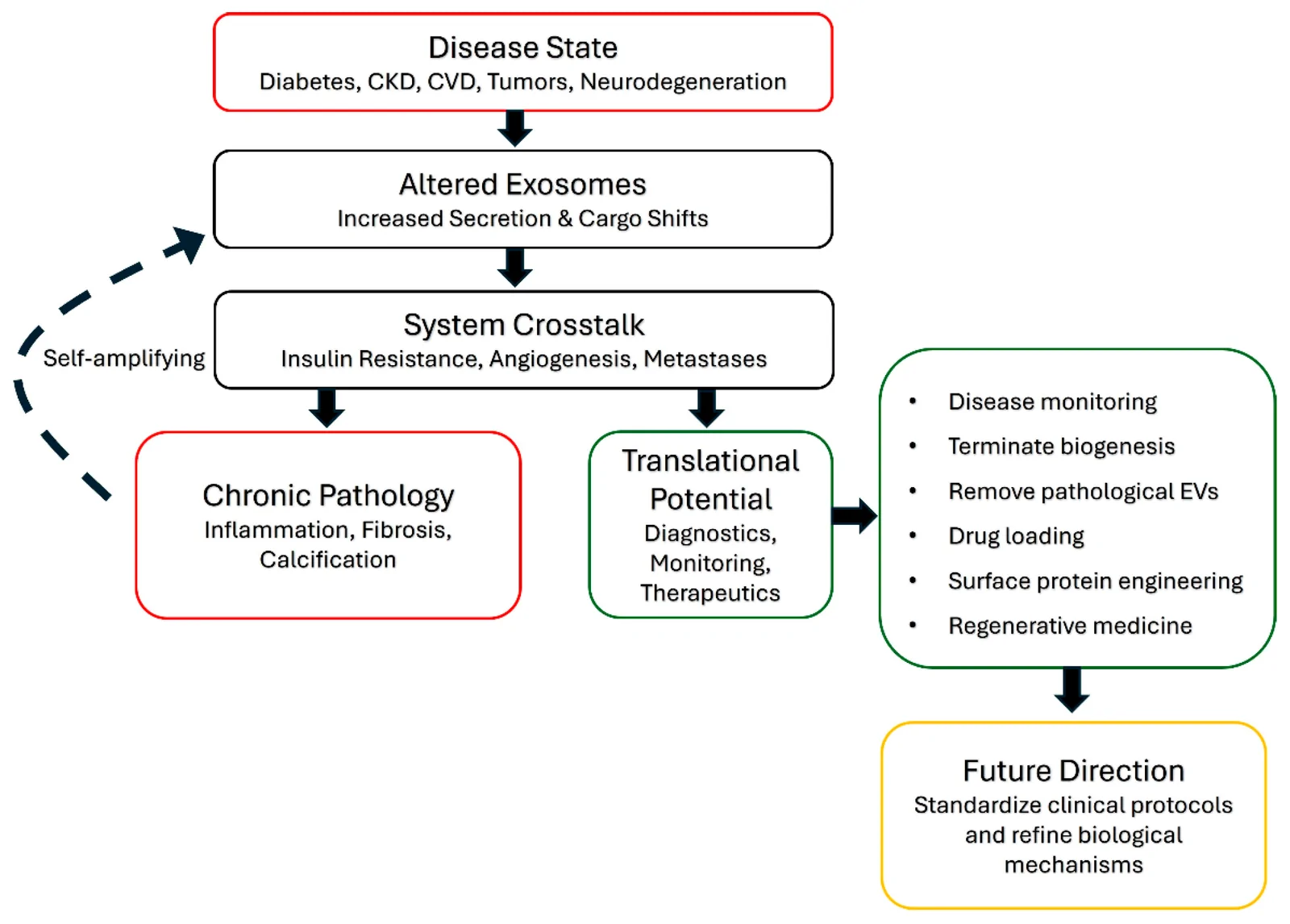
Conceptual overview of exosome-mediated disease progression and clinical translation. Altered exosome secretion and cargo drive chronic pathology while providing opportunities for diagnostics, therapeutics, and regenerative medicine.

In summary, this review describes the molecular mechanisms underlying exosome biogenesis, cargo selection, functional heterogeneity, and inter-organ communication, and their role in disease progression (Figure 4). As presented, exosomes present as active signaling platforms that modulate local and systemic communication through proteins, lipids, metabolites as well as nucleic acids. We discussed pathological conditions including diabetes, chronic kidney disease, cardiovascular diseases, neurodegenerative disorders, and cancer that alter exosome biogenesis and their molecular cargo, leading to inflammation, oxidative stress, fibrosis, metabolic dysfunction, vascular damage, immune dysregulation, and neurodegeneration in different organs. Furthermore, we also demonstrated the recent advances in the potential of exosomes as minimally invasive biomarkers and therapeutic agents. Rather than considering these processes as isolated biological events, this review presents an integrated framework linking exosome biogenesis, cargo remodeling, and inter-organ communication to the coordinated progression of systemic diseases. We believe that the studies consolidated in this review illustrate the molecular mechanisms related to exosome biogenesis, cargo selection and target-cell interactions that establish exosomes as active mediators of systemic signaling and support their application as diagnostic biomarkers and therapeutic targets. Continued research into exosome biology, the molecular mechanisms regulating exosome function, and standardized experimental methodologies will further advance the field and facilitate the translation of exosome-based approaches into clinical practice.

## Figures and Tables

**Table 1:** Principal mechanisms underlying exosome-mediated signaling and their biological consequences.

Signaling Mechanism	Major Mediators	Representative Signaling Pathways	Major Biological Consequences	Reference
Recipient-cell interaction	PD-L1	PD-1	Immune evasion	[15,95,98,99,100,101,102,103,104]
	Syntaxin-4; SNAP-23; VAMP-7	Membrane fusion	Cargo delivery	
	DNMT mRNAs	Genome-wide DNA hypermethylation	Epigenetic regulation	
	miR-1271-5p	SOX6 repression	Cardiomyocyte survival	
Signal transduction	Microglial exosomal miR-155-5p	NF-κB	Inflammation	[105,107,108,109,110,111,112,113]
	miR-143-3p	PI3K/AKT	M1 macrophage polarization	
	miR-486-5p	PI3K/AKT	Cardiomyocyte survival	
	HMGB1	TLR4/NF-κB/STAT3	Tumor progression; immune suppression	
	miR-21	STAT3	Chronic inflammatory signaling	
Inflammatory amplification	miR-30b-5p	NF-κB	Chronic inflammation	[7,115,117,118,119,120,127]
	SHH	Hormone-sensitive lipase signaling	Insulin resistance	
	RBP4	TNF-α; IL-6	Macrophage activation	
Metabolic regulation	Adipose tissue macrophage-derived exosomal miR-155	SOCS1	Insulin resistance	[121,122,123,124,125,126]
	miR-34a	M2 macrophage polarization	Metabolic inflammation	
	NOX2; NOX4	MEK1/2-ERK1/2	Oxidative stress	
	Palmitic acid; stearic acid	TLR4	Impaired insulin sensitivity	
	Nutrient availability	mTORC1-SCYL1	Regulation of exosome secretion	

The same mediator may be listed in more than one mechanistic context when it regulates distinct signaling pathways or biological responses. Abbreviations: HMGB1, high mobility group box 1; IL, interleukin; NF-κB, nuclear factor kappa-light-chain-enhancer of activated B cells; NLRP3, NLR family pyrin domain containing 3; NOX, NADPH oxidase; PD-1, programmed cell death protein 1; PD-L1, programmed death-ligand 1; PI3K/AKT, phosphoinositide 3-kinase/protein kinase B; RBP4, retinol-binding protein 4; SHH, Sonic hedgehog; SNARE, soluble N-ethylmaleimide-sensitive factor attachment protein receptor; SOCS1, suppressor of cytokine signaling 1; STAT3, signal transducer and activator of transcription 3; TGF-β, transforming growth factor-beta.

**Table 2:** Clinical applications of exosomal biomarkers in human diseases.

Clinical Application	Biological Basis	Current Evidence	Major Limitation	Translational Readiness	References
Biomarkers & Diagnostics	Exosome- and EV-associated cargo reflects tissue pathophysiology	Human studies	Limited analytical standardization	Early clinical/clinical validation	[215,216,217,218,228,230]
Prognostic Value	Exosomal cargo correlates with disease outcome	Human studies	Limited prospective validation	Early clinical	[235,236]
Disease Monitoring	Dynamic changes in exosomal cargo reflect disease progression	Human studies	Limited analytical standardization	Early clinical	[238,239]
Plasma Exosomes & Systemic Disease	Exosome-mediated systemic signaling	Mechanistic and animal studies	Complex biodistribution and rapid organ uptake	Preclinical	[116,121,237]
Long-Term Stability	Protection of exosomal cargo by lipid bilayers	Human studies	Limited analytical standardization	Early clinical	[240]
Cargo Loading & Sorting	Selective cargo loading and sorting	Mechanistic studies	Limited mechanistic understanding and reproducibility	Preclinical	[71,241]
Therapeutic Use of Endogenous Exosomes	Activation of cytoprotective signaling pathways	Animal studies	Limited clinical validation and mechanistic understanding	Preclinical	[242]
MSC-Derived Regenerative Therapy	Transfer of regenerative bioactive molecules	Preclinical studies	Lack of standardized clinical protocols	Preclinical/early clinical	[243,244,245]
Engineered Drug Delivery	Surface engineering for targeted drug delivery	Proof-of-concept animal studies	Limited target efficiency, scalable manufacturing, and off-target biodistribution	Preclinical	[246,247,248]
Drug Loading	Loading of therapeutic cargo into exosomes and EVs	Preclinical models	Limited loading efficiency, cargo retention, and reproducibility	Preclinical	[249,250,251]
Gene Therapy	Delivery of therapeutic nucleic acids	Preclinical studies	Limited cargo loading efficiency and tissue specificity	Preclinical	[252,253,254]

Abbreviation: EVs, extracellular vesicles.

## Data Availability

The original contributions presented in this study are included in the article. Further inquiries can be directed to the corresponding author.

## Abbreviations

The following abbreviations are used in this manuscript:

EVs	extracellular vesicles
miRs	microRNAs
EEs	early endosomes
ILVs	intraluminal vesicles
MVBs	multivesicular bodies
ESCRT	endosomal Sorting Complex Required for Transport
MHC	major histocompatibility complex
ALIX	aLG-2 interacting protein X
PA	phosphatidic acid
Cav-1	Caveolin-1
DAMPs	damage-associated molecular patterns
NF-κB	nuclear factor kappa-light-chain-enhancer of activated B cells
SNARE	soluble N-ethylmaleimide-sensitive agent-binding protein
SOX6	SRY-box 6
PI3K/AKT	phosphoinositide 3-kinase/protein kinase B
PTEN	phosphatase and tensin homolog deleted on chromosome 10
STAT3	transcription factor 3
HMGB1	high mobility group box 1
TLR4	toll-like receptor 4
TGF-β	transforming growth factor-beta
NLRP3	NLR family inflammasome containing the pyrin domain 3
TNF-α	tumor necrosis factor alpha
IL	interleukin
Shh	sonic hedgehog
RBP4	retinol-binding protein 4
CKD	chronic kidney disease
SOCS1	suppressor of cytokine signaling 1
NOX	NADPH oxidase
MEK1/2	mitogen-activated protein kinase 1 and 2
ERK1/2	extracellular signal-regulated kinases 1 and 2
mTORC1-SCYL1	mammalian target of rapamycin complex 1- SCY1-like pseudokinase 1
AGE-RAGE	advanced glycation end-product–receptor for advanced glycation end-products
ROS	reactive oxygen species
FOXP1	forkhead box P1
IS	indoxyl sulfate
GAG	glycosaminoglycan
MMP1	matrix metallopeptidase 1
CTSL	cathepsin L
ICAM-1	intercellular adhesion molecule 1
VCAM-1	vascular cell adhesion molecule-1
OPN	β-catenin-osteopontin
TNFAIP8	tumor necrosis factor alpha-induced protein 8
CVD	cardiovascular disease
mTOR	mechanistic target of rapamycin
HIF-1α	hypoxia-inducible factor 1-alpha
Hsps	heat shock proteins
DUSP5	dual-specificity protein phosphatase 5
MAPK	mitogen-activated protein kinase
KLF4	krüppel-like factor 4
EGFR	epidermal growth factor receptor
IKK2	IκB kinase β
PCSK8	proprotein convertase subtilisin/kexin type 8
VSMC	vascular smooth muscle cells
EndMT	endothelial–mesenchymal transition
LPS	lipopolysaccharide
TDEs	tumor-derived exosomes
GDF-15	growth and differentiation factor 15
NK	natural killer
Treg	regulatory T cell
MDSC	myeloid-derived suppressor cell
CSF	cerebrospinal fluid
pHERV-W ENV	human endogenous retrovirus type W exosomal envelope protein
LPPs	lipoproteins
AMPK/PGC-1α	AMP-activated protein kinase–peroxisome proliferator-activated receptor gamma coactivator 1-alpha
